# Development and psychometric evaluation of the diabetic Men’s dietary behaviors inventory based on the theory of reasoned action

**DOI:** 10.1186/s13690-018-0328-7

**Published:** 2019-01-17

**Authors:** Fataneh Goodarzi, Marzieh Araban, Ahmad Ali Eslami, Fereshteh Zamani-Alavijeh

**Affiliations:** 1 School of Health, Isfahan University of Medical Sciences, Isfahan, Iran; 2 Social Determinants of Health Research Center, Department of Health Education and Promotion , Public Health School, Ahvaz Jundishapur University of Medical Sciences, Ahvaz, Iran; 3 Department of Health Education and Promotion, School of Health, Isfahan University of Medical Sciences, Hezarjarib Street, Isfahan, Iran

**Keywords:** Diabetes mellitus, Dietary behaviors, Psychometric evaluation, Instrument development, Methodological study

## Abstract

**Background:**

Unhealthy dietary behaviors have progressively increased the prevalence of diabetes mellitus. Thus, assessing such behaviors and their associated beliefs by valid measurement tools seems essential. This study sought to develop and evaluate the psychometric properties of the Diabetic Men’s Dietary Behaviors Inventory based on the Theory of Reasoned Action.

**Methods:**

Initially, a preliminary 78-item inventory on diabetic men’s dietary beliefs and behaviors was developed based on the six constructs of the Theory of Reasoned Action. Then, psychometric evaluation methods were employed to select the most appropriate items and also to validate the inventory. The validity of the inventory was assessed through face, content, and construct validity assessment. For construct validity assessment, a sample of 206 diabetic men was selected from two educational, research and healthcare settings located in Isfahan, Iran. The inventory was completed for all men through interviewing them. The reliability of the inventory was evaluated through internal consistency assessment.

**Results:**

The preliminary inventory contained 78 items, 33 of which were excluded during the phases of psychometric evaluation. Exploratory factor analysis revealed a five-factor structure for the inventory; the factor loads ranged from 0.41 to 0.80. All items were significantly correlated with the inventory. Cronbach’s alpha values of all factors were greater than 0.6, denoting the high internal consistency of the inventory.

**Conclusion:**

The Diabetic Men’s Dietary Behaviors Inventory is a valid and reliable instrument for evaluating diabetic men’s dietary perceptions and behaviors.

## Introduction

Diabetes mellitus (DM) is among the most prevalent metabolic disorders [1] which causes around four million deaths and many disabilities each year [2, 3]. The number of 20–79 year-old diabetic patients is estimated to reach 642 million by 2040 [4]. The prevalence of DM among Iranian adults was reported to be about 2–10% [5]. According to the recent reports, about 9.6% of Iranian men suffer from DM [6]. Although the prevalence of DM among Iranian men is lower than their female counterparts [7], the prevalence is progressively increasing [8]. Moreover, in Iran, pre-diabetes is more prevalent among men than women [9].

DM and its complications significantly affect the quality of life of the afflicted patients and their families [10]. Moreover, compared with healthy people, hypertension and renal disorders are respectively two and seventeen times more prevalent among diabetics [11]. Not only adults, but also the youth are afflicted by DM. DM-related costs are around 10% of the total healthcare costs [10]. Furthermore, diabetic and pre-diabetic people are more at risk for developing cancer [12].

According to the World Health Organization, one of the main components of DM management is engagement in self-care activities, particularly dietary modifications [13–16]. Nonetheless, investigations show limited engagement in healthy dietary behaviors among patients with chronic conditions such as DM [15, 17–19]. Successful DM management greatly depends on self-care activities such as having a healthy diet and performing adequate physical activity [17, 20].

Patients’ engagement in healthy behaviors greatly depends on their beliefs [20]. In other words, one of the factors behind patients’ poor adherence to their dietary regimens is their beliefs [20–22]. Studies show that beliefs and perceptions of the outcomes of behaviors can predict dietary behaviors [23]. Moreover, diabetics’ perceptions of their family members’ ideas can motivate them for healthy eating and improve their dietary behaviors [16, 24]. Thus, a prerequisite to any intervention for improving their dietary behaviors is to assess and identify beliefs and perceptions contributing to such behaviors [17]. Some studies showed that beliefs and attitudes cannot always predict behaviors and thus, it is needed to use appropriate theories to assess all factors contributing to behaviors.

Health education theories and models can play a pivotal role in identifying factors contributing to behaviors. One of these theories is the Theory of Reasoned Action (TRA) [25, 26].TRA integrates both behavioral and normative beliefs [25] and holds that the most significant factor behind behaviors is intention. In other words, behavioral intention is the most significant predictor of behaviors. Behavioral intention, in turn, is determined by two factors, namely attitude toward behavior and subjective norms. These two factors are affected by personal beliefs. Attitude consists of behavioral beliefs and outcome evaluation while subjective norms comprises normative beliefs and motivation to comply [27].

It has been reported that knowledge and beliefs towards a certain behavior may be different among males and females. In this regard, the results of earlier reports shows that beliefs and behavior of men with diabetes are different than that of women with diabetes [28, 29]. Men do less attention to nutritional education than women and the taste of food play an important role in their food choices [30, 31], additionally, also it is less probable for men than women to control their weight and adhere with healthy foods [32]. As such, we cannot use the same measure for women as for men with diabetes. Although the necessity to assess diabetic men’s dietary beliefs and behaviors [23, 33], there is currently no gender-specific tool for assessing diabetic men’s dietary behaviors inventory. The present aimed at developing and evaluating the psychometric properties of the Diabetic Men’s Dietary Behaviors Inventory (DMDBI).

## Methods

This cross-sectional study was carried out in 2018. The study was replicated on an earlier study sample, [34] with a time interval of 12 months, attending the Isfahan Diabetes Clinic.

### Scale development

The constructs of TRA (i.e. behavioral beliefs, outcome evaluation, normative beliefs, motivation to comply, behavioral intention, and dietary behaviors) were used to develop an item pool; the recommendations of questionnaire construction based on TRA as stated by Fishbein were considered for developing the statements. The pool was developed in six dimensions through doing a literature review [35] and seeking the comments of a panel of three health education specialists, two nutritionists, one nurse, and one biostatistician. The items were worded carefully in order to minimize ambiguities and enhance readability. In total, the item pool comprised 78 items on perceptual factors affecting diabetic men’s dietary behaviors. The items of the “Dietary behaviors” dimension were scored from 1 (“Almost Never”) to 5 (“Almost always”) while the other items were scored on a five-point Likert-type scale as follows: 1: “Strongly agreed”; 2: “Agreed”; 3: “No opinion”; 4: “Disagreed”; and 5: “Strongly disagreed”. Higher scores on the inventory reflected more positive behavioral beliefs and higher likelihood to show healthy dietary behaviors. Some items had been worded negatively and thus, were scored reversely.

### Face validity assessment

Face validity of the DMDBI was assessed qualitatively. Accordingly, face-to-face personal interviews were held with thirty diabetic men, who were independent from the study sample, in order to seek their comments on the wording of the items and general appearance of the inventory. The items were amended according to their comments.

### Content validity assessment

The content validity of the DMDBI was assessed through both quantitative and qualitative methods. During qualitative content validity assessment, twelve experts were interviewed and asked to comment on the wording, readability, and comprehensibility of the items, the soundness of the dimensions and item allocation, and the appropriateness of the scoring system. The inventory was amended based on their comments. For quantitative content validity assessment, ten other experts were invited to determine whether each DMDBI item was “essential”, “useful but not essential”, or “not essential”. Their comments were used to calculate Content Validity Ratio (CVR). According to Lawshe (1975), items with a CVR of less than 0.62 were excluded [36]. After that, the same experts were asked to evaluate the relativity, simplicity, and clarity of the items by using a four-point Likert-type scale from 1 to 4. For instance, the clarity of each item was evaluated as follows: 1: “Not clear”; 2: “Relatively clear”; 3: “Clear”; 4: “Completely clear”. Then, Content Validity Index (CVI) was calculated for each item through dividing the number of experts who had scored that item 3 or 4 by the total number of the experts [37]. Items with a CVI of higher than 0.79 were considered to have acceptable content validity [7].

### Construct validity assessment

Classical item analysis and exploratory factor analysis were performed to determine the factor structure and evaluate the construct validity of the inventory. In this phase, a convenient sample of 206 diabetic men was selected from two educational, research and healthcare settings affiliated to Isfahan University of Medical Sciences, Isfahan, Iran. They were included if they were older than 30, suffered from DM for more than six months (as diagnosed by DM specialists), lived in Isfahan, had basic literacy skills, and voluntarily agreed to participate in the study. Primarily, they completed, in fifteen minutes, a questionnaire containing items on their demographic characteristics, height, and weight. Then, the Persian DMDBI was completed through holding twelve-minute personal interviews with the participants. Both interviewer and interviews spoke Persian and thus, the results of the study were translated into English and reported in the present article. Thereafter, the collected data were used to perform classical item analysis and exploratory factor analysis via the SPSS software.

#### Classical item analysis

Item analysis was performed by calculating the Corrected Item-Total Correlation (CITC). Items with a CITC of higher than 0.3 were considered as appropriate [38]. Moreover, the normality and skewness of the distribution of each item were assessed. The minimal acceptable value for skewness was 1.96.

#### Exploratory factor analysis

Primarily, the Kaiser-Meyer-Olkin (KMO) test and the Bartlett’s test of sphericity were done to assess the adequacy of the sample and the appropriateness of factor analysis model. Then, exploratory factor analysis with varimax rotation, a cutoff point of 0.4 for factor load, and an Eigenvalue of higher than 1 was performed to identify the main factors of the inventory. Identified factors were confirmed through using the scree plot [39, 40].

### Reliability assessment

The reliability of the inventory was evaluated through internal consistency assessment by calculating the Cronbach’s alpha of the total inventory and its dimensions.

### Ethics assessment

In this study, we attempted to strictly adhere to the principles of Ethics for Medical Research. Accordingly, we primarily obtained an ethical approval for the study from the Ethics Committee of Isfahan University of Medical Sciences with ethics number: IR.MUI.REC.1396.1.196. Moreover, we secured participants’ informed consent for participation in the study and ensured them that their personal data would remain confidential.

## Results

The means of participants’ age and body mass index were 59.26 ± 9.74 and 26.70 ± 4.34, respectively. Table 1 shows participants’ demographic characteristics.

**Table 1:** Participants’ demographic characteristic (*N* = 206)

	Minimum	Maximum	Mean	Std. Deviation
Time from diagnosis (Year)	0.50	45.00	11.08	8.50
Body mass index (kg/m^2^)	17.63	41.95	26.70	4.34
Weight(kg)	40.00	127.00	78.88	14.15
Age (years)	34.00	89.00	58.26	9.74
Educations (years)	5	16	8	2.78

The preliminary DMDBI contained 78 items in six dimensions namely behavioral beliefs (19 items), outcome evaluation (8 items), normative beliefs (17 items), motivation to comply (18 items), behavioral intention (six items), and dietary behaviors (10 items).

### The results of quantitative content validity assessment

During the process of quantitative content validity assessment, fifteen items were excluded due to a CVI of less than 0.79. Moreover, three items obtained a CVR of less than 0.62 and were excluded. Thus, 60 items remained in the inventory—11, 8, 13, 12, 6, and 10 items in the aforementioned dimensions, respectively.

### The results of CITC analysis

The 60-item DMDBI was then subjected to CITC analysis during which, 27 items were excluded due to acquiring a CITC of less than 0.3 or a skewness value of less than 1.96. Hence, 33 items remained in the inventory (Table 2).

**Table 2:** Corrected Item-Total Correlation of the Inventory

Factor	Item	Mean)Std. Deviation)	Corrected Item-Total Correlation	Skewness	Std. Error of Mean
Behavioral beliefs	Substituting solid cooking oil with liquid oils (such as canola or olive) has no significant effects on maintaining my health.	1.7 (0.86)	0.40	0.44	0.05
	Consuming high-fat dairy products (such as milk, yoghurt, and cheese) helps maintain my health.	1.9 (0.85)	0.59	0.02	0.05
	Consuming mayonnaise sauce is beneficial to my health.	1.8 (0.83)	0.52	0.20	0.05
Outcome evaluation	Controlling my blood sugar is the most important thing for me.	2.0 (0.82)	0.60	−0.06	0.05
	Preventing nighttime blood sugar decline is really vital to me.	2.0 (0.78)	0.58	−0.03	0.05
	Enhancing the resistance of my body to infections and diseases is of great importance for me.	2.1 (0.77)	0.70	−0.27	0.05
	Postponing the complications of diabetes mellitus is of great importance for me	2.0 (0.74)	0.65	−0.10	0.05
	Maintaining my health is the most important thing for me.	2.1 (0.69)	0.73	−0.26	0.04
	Decreasing the risk for developing the complications of diabetes mellitus is very important for me.	2.1 (0.73)	0.71	−0.16	0.05
	Preventing cardiovascular diseases is very important for me.	2.0 (0.63)	0.81	−0.06	0.04
Normative beliefs	My family members and significant others nod their heads in agreement when I drink soft drinks and processed juices.	1.8 (0.83)	0.65	0.21	0.05
	My family members and significant others disagree with me in using liquid cooking oil (such as canola or olive) instead of solid oil	1.8 (0.81)	0.72	0.32	0.05
	My family members and significant others nod their heads in agreement when I consume fried and high-fat foods.	1.8 (0.81)	0.77	0.21	0.05
	My family members and significant others disagree with me in separating fat from red meat and chicken from its skin.	1.9 (0.80)	0.80	0.14	0.05
	My family members and significant others disagree with consuming low-fat dairy products.	1.8 (0.80)	0.74	0.30	0.05
	My family members and significant others express their disagreement when I use lemon juice, vegetables, and low-fat yoghurt instead of sauce.	1.9 (0.80)	.0.67	0.11	0.05
	My family members and significant others approve me when I use packed processed juices.	1.8 (0.85)	0.67	0.34	0.05
Motivation to comply	I care about the opinion of my family members and significant about consuming low-fat dairy products is important for me.	2.0 (0.77)	0.40	−0.06	0.05
	I tend to follow the opinion of my family members and significant others about the type of oil to consume.	1.9 (0.78)	0.40	0.07	0.05
	The opinion of my family members and significant others opinion about consuming fried and high-fat foods is important for me.	1.8 (0.76)	0.37	0.24	0.05
	I tend to follow the opinion of my family members and significant others about fish consumption.	1.9 (0.70)	0.39	0.13	0.04
	I wouldn’t consume simmered foods if my family members and significant others do not like them.	1.8 (0.78)	0.32	0.20	0.05
	I tend to follow the opinion of my family members and significant others about the consumption of fruits, vegetables, and grains.	1.8 (0.76)	0.38	0.21	0.05
Intention	In the next month, I intend to eat three main meals a day as recommended by my doctor.	1.9 (0.70)	0.39	0.02	0.04
	In the next month, I intend to eat three snacks a day as recommended by my doctor	1.8 (0.72)	0.31	0.19	0.05
	In the next month, I intend to eat main meals and snacks at predetermined times as recommended by my doctor.	1.8 (0.75)	0.67	0.34	0.05
	In the next month, I intend to eat a diversified diet and all food groups as recommended by my doctor.	1.8 (0.76)	0.55	0.25	0.05
Dietary behaviors	I eat low-salt foods.	1.9 (0.75)	0.63	0.13	0.05
	For cooking foods, I use liquid oil (canola or olive) instead of solid oil.	1.8 (0.77)	0.54	0.25	0.05
	I avoid consuming fried and high-fat foods.	1.8 (0.76)	0.41	0.29	0.05
	Before cooking, I separate fats from white and red meats.	1.9 (0.78)	0.32	0.13	0.05
	I use low-fat dairy products.	1.8 (0.74)	0.31	0.31	0.05
	I avoid consuming packed processed juices.	1.8 (0.79)	0.32	0.35	0.05

### The results of exploratory factor analysis

The KMO test value was 0.81, which implies that the study sample was adequate. Moreover, the result of Bartlett’s test of sphericity was significant (2285.67; *P* value < 0.001) and thus, the factor analysis model was appropriate. Exploratory factor analysis yielded nine factors with an Eigenvalue of greater than 1. However, some of the extracted factors were weak, contained just one or two items, and were not compatible with the theoretical assumptions of the study. Thus, factor analysis was redone by using an Eigenvalue of 1.2 which revealed a five-factor structure for the DMDBI. This factor structure was greatly similar to the theoretical assumptions of the study. The factor loads of the factors ranged between 0.41 and 0.80 and the total variance of the five-factor model was 47%. The factors were labeled based on their corresponding items and the existing literature. The variances and the factor load ranges of the DMDBI factors were respectively as follows: **“Beliefs about behavioral outcomes”**: 19.65% and 0.55–0.76; **“Outcome evaluation”**: 11.67 and 0.62–0.80; **“Dietary behaviors”**: 6.97% and 0.41–0.69; **“Motivation to comply”**: 4.69% and 0.49–0.62; and “**Intention to adjust and diversify dietary regimen”**: 3.98% and 0.47–0.69 (Table 3).

**Table 3:** The results of factor analysis and internal consistency assessment (Cronbach’s alpha)

Item	Factor 1	Factor 2	Factor 3	Factor 4	Factor 5
	Beliefs about behavioral outcomes	Outcome evaluation	Dietary behaviors	Motivation to comply	Intention to adjust and diversify dietary regimen
Substituting solid cooking oil with liquid oils (such as canola or olive) has no significant effects on maintaining my health.	0.55				
Consuming high-fat dairy products (such as milk, yoghurt, and cheese) helps maintain my health.	0.58				
Consuming mayonnaise sauce is beneficial to my health.	0.54				
My family members and significant others nod their heads in agreement when I drink soft drinks and processed juices.	0.72				
My family members and significant others disagree with me in using liquid cooking oil (such as canola or olive) instead of solid oil	0.74				
My family members and significant others nod their heads in agreement when I consume fried and high-fat foods.	0.74				
My family members and significant others disagree with me in separating fat from red meat and chicken from its skin.	0.78				
My family members and significant others disagree with consuming low-fat dairy products.	0.72				
My family members and significant others express their disagreement when I use lemon juice, vegetables, and low-fat yoghurt instead of sauce.	0.71				
My family members and significant others approve me when I use packed processed juices.	0.76				
Controlling my blood sugar is the most important thing for me.		0.62			
Preventing nighttime blood sugar decline is really vital to me.		0.63			
Enhancing the resistance of my body to infections and diseases is of great importance for me.		0.68			
Postponing the complications of diabetes mellitus is of great importance for me		0.70			
Maintaining my health is the most important thing for me.		0.69			
Decreasing the risk for developing the complications of diabetes mellitus is very important for me.		0.70			
Preventing cardiovascular diseases is very important for me.		0.80			
I eat low-salt foods.			0.64		
For cooking foods, I use liquid oil (canola or olive) instead of solid oil.			0.50		
I avoid consuming fried and high-fat foods.			0.47		
Before cooking, I separate fats from white and red meats.			0.69		
I use low-fat dairy products.			0.41		
I avoid consuming packed processed juices.			0.42		
I care about the opinion of my family members and significant about consuming low-fat dairy products is important for me.				0.62	
I tend to follow the opinion of my family members and significant others about the type of oil to consume.				0.56	
The opinion of my family members and significant others opinion about consuming fried and high-fat foods is important for me.				0.60	
I tend to follow the opinion of my family members and significant others about fish consumption.				0.49	
I wouldn’t consume simmered foods if my family members and significant others do not like them.				0.51	
I tend to follow the opinion of my family members and significant others about the consumption of fruits, vegetables, and grains.				0.58	
In the next month, I intend to eat three main meals a day as recommended by my doctor.					0.56
In the next month, I intend to eat three snacks a day as recommended by my doctor					0.47
In the next month, I intend to eat main meals and snacks at predetermined times as recommended by my doctor.					0.69
In the next month, I intend to eat a diversified diet and all food groups as recommended by my doctor.					0.60
Eigenvalue	6.48	3.85	2.30	1.55	1.31
Variance%	19.65	11.67	6.97	4.69	3.98
Factor Loading Range	0.55–0.76	0.62–0.80	0.41–0.69	0.49–0.62	0.47–0.69
Cronbach’s Alpha	0.66	0.83	0.89	0.64	0.73

The first factor, i.e. **“Beliefs about behavioral outcomes”**, contained ten items, seven items of which were related to normative beliefs while the remaining three items dealt with behavioral beliefs. Thus, this factor showed individuals’ perceptions of the outcomes of behaviors on their own health and also on others’ reactions. The second factor was labeled **“Outcome evaluation”** and was completely congruent with the corresponding construct of TRA. The number of items of this factor was seven. The third factor was **“Dietary behaviors”** and contained six items. The items of this factor were related to dietary behaviors. This factor reflected an individual’s attempts to abstain from using unhealthy foods namely high-salt and high-fat foods. The fourth factor included six items and was labeled **“Motivation to comply”**. Thus, this factor was compatible with the motivation to comply construct of TRA. The last factor was labeled **“Intention to adjust and diversify dietary regimen”**. It included four items on behavioral intention and showed an individual’s decision to adjust and diversify his dietary regimen (Table 3).

### The results of reliability assessment

After validity assessment, the reliability of the DMDBI was assessed through evaluating its internal consistency. The Cronbach’s alpha of the inventory was 0.86 and the Cronbach’s alpha values of its dimensions ranged between 0.66–0.89 (Table 3).

## Discussion

The present study sought to develop and evaluate psychometric properties of the DMDBI based on TRA.In order to accomplish this aim, a preliminary 78-item inventory was developed based on the six constructs of TRA. Then, CVI and CVR were calculated and 60 items with acceptable CVI and CVR were kept. After that, item analysis was performed which yielded a 33-item inventory. Finally, exploratory factor analysis revealed a five-factor structure for the inventory. The extracted factors were remarkably similar to the theoretical underpinnings of the study, i.e. the constructs of TRA. TRA holds that **“intention”** results in **“behavior”** and is affected by “attitude”. “Attitude”, in turn, consists of **“behavioral beliefs”** and **“evaluation of behavioral outcomes”**. Another construct of the theory is “subjective norms” which affects “behavioral intention”. The “subjective norms” construct comprises **“normative beliefs”** and **“motivation to comply”**. However, the factor structure of the 33-item DMDBI was slightly different from the constructs of TRA. Internal consistency analysis and Cronbach’s alpha values revealed the acceptable reliability of the DMDBI.

The first factor of the DMDBI was **“Beliefs about behavioral outcomes”**. The items loaded into this factor were extracted from the **“behavioral beliefs”** and **“normative beliefs”** constructs of TRA. Thus, this factor denotes an individual’s beliefs about the outcomes of a dietary behavior [41] and also about significant others’ reactions to the behavior [41, 42]. The second factor of the inventory was **“Outcome evaluation”**. This factor is the second component of the “attitude” construct of TRA and denotes an individual’s evaluation of healthy dietary behaviors outcomes. As a determining factor behind attitude, this factor is a significant predictor of behavioral intention. Thus, an individual’s attitude to a behavior can motivate him/her to show or modify that behavior. Aghamolaei et al. (2005) and Polly (1992) also confirmed that health-related beliefs are significantly correlated with diabetics’ self-care activities [43, 44]. Besides, Didarloo et al. (2011) [42]. Reported a significant positive correlation between attitude and behavioral intention these findings imply that people who hold stronger beliefs about self-care activities are more likely to engage in the activities.

The third factor of the DMDBI was **“Dietary behaviors”** which shows an individual’s abstinence from using unhealthy foods. Dietary behaviors have a pivotal role in preventing and managing DM and its complications [45–47]. TRA-based DMDBI can be used to assess diabetic patients’ dietary behaviors and subsequently, develop strategies for modifying such behaviors and improving patient health [47]. The fourth factor extracted from the DMDBI was **“Motivation to comply”**. The items of this factor represent the motivation for considering the opinions of family members and significant othersin the time of decision making about dietary behaviors. This factor is completely congruent with the “motivation to comply” construct of TRA. Other people can affect the decisions about healthy or unhealthy behaviors. In other words, one may base his/her intention on the wills of others [42]. Our findings with regard to the “Motivation to comply” factor was congruent with the findings of previous studies. For example, Didarloo et al. (2011) made a cross-sectional study on 352 diabetic women and found that compared with people who did not experience social pressure, those who were under the pressure of their significant others were more likely to show an intended behavior. Moreover, they reported that through affecting intention, subjective norms contribute to the exhibition of self-care behaviors [42]. Nonetheless, Gholami et al. (2014), Kim (2003), and Stoel (2009) reported no significant correlation between subjective norms and the intention to consume fruits and eat a healthy diet [48, 49]. It seems that the relationship between intention and subjective norms widely varies according to the types of behaviors [50, 51]. The last factor of the DMDBI was **“Intention to adjust and diversify dietary regimen”**. This factor is relatively consistent with the “behavioral intention” construct of TRA which consists of thinking and deciding about showing a behavior [26]. Some previous studies also assessed the intention to adjust and diversify dietary regimen. Stoel (2009) found a significant correlation between attitude and behavioral intention in that people with more positive attitudes towards a diversified diet were more healthy and thus, more likely to show healthy behaviors. TRA holds that intention is the direct and essential prerequisite for behavior [25, 52]. Some of the previous studies also dealt with the intention to abstain from high-risk behaviors such as unhealthy eating [53] and smoking [54]. These studies indicated that the intention to abstain was significantly correlated with behavior, attitude, and subjective norms. Gholami et al. (2014) also noted that dietary habits and attitudes can affect the intention to consume fruits and vegetables [48].

The findings of the present study showed that the DMDBI has acceptable validity and reliability and can be used to study diabetic men’s dietary perceptions and beliefs. Exploratory factor analysis revealed that all DMDBI items had great construct validity. The factor loads of all five factors of the inventory were greater than 0.4, denoting acceptable factor load. Factor load values that are greater than 0.4 show that all items are important and the inventory has acceptable validity. The reliability of the DMDBI was also assessed through the internal consistency assessment method. Cronbach’s alpha values of three DMDBI factors were greater than 0.7, denoting high internal consistency while two factors had acceptable Cronbach’s alpha values (higher than 0.6).

### Limitation and direction for future researches

One of the limitations of the study was that confirmatory factor analysis was not performed for assessing the DMDBI items constructs. Further studies are recommended to assess construct validity of DMDBI. Moreover, study sample was rather small and consisted only of men. Thus, the findings should be generalized cautiously.

## Conclusion

Developed in the present study, the DMDBI was shown to be a valid and reliable instrument for measuring diabetic men’s dietary perceptions and behaviors. Thus, the inventory can be used in studies to assess the effects of nutrition educational interventions on men’s with diabetes. The usefulness of the inventory for female populations should be assessed in future studies.

## Abbreviations

CVR: Content validity ratio.
CVI: Content validity index.
EFA: Explanatory factor analysis.
